# Descriptive analysis of a SARS-CoV-2 outbreak among health-care workers in a regional hospital in the Philippines

**DOI:** 10.5365/wpsar.2023.14.3.1050

**Published:** 2023-09-30

**Authors:** Lily Anne N Safilo, Ray Justin C Ventura, Mariz Zheila C Blanco, Karen B Lonogan, Rosario P Pamintuan, Rio L Magpantay

**Affiliations:** a Provincial Health Office, Mountain Province, Philippines.; b Department of Health, Manila, Philippines.; c Philippines Field Epidemiology Training Programme, Department of Health, Manila, Philippines.

## Abstract

**Objective:**

On 25 July 2022, trainees from the Field Epidemiology Training Programme in Northern Luzon, Philippines were sent to conduct an epidemiological investigation of six confirmed cases of severe acute respiratory syndrome coronavirus 2 (SARS-CoV-2) among staff of a regional hospital in Mountain Province. The investigation had three objectives: to profile the cases, identify the source and mode of transmission, and recommend prevention and control measures.

**Methods:**

Descriptive epidemiology was used to investigate the outbreak, with the standard case definition issued by the Philippine Department of Health.

**Results:**

A total of 167 hospital personnel and interns tested positive for SARS-CoV-2 infection between 6 July and 31 August 2022, with a peak in the number of cases on 20 July. Among the cases, 57 (34%) had a history of travel, with 41 (25%) having travelled to Boracay island to attend team-building activities. Most cases were asymptomatic, and the most affected group was those aged 30–34 years. The highest number of cases occurred among nurses. It was discovered that the team-building activities on Boracay did not strictly adhere to safety protocols.

**Discussion:**

This outbreak suggests that transmission of SARS-CoV-2 among health-care workers can occur through contact with other staff members outside of the hospital setting and highlights the importance of strict adherence to safety protocols to prevent the spread of SARS-CoV-2.

On 12 July 2022, the Provincial Epidemiology and Surveillance Unit in Mountain Province, Philippines, reported six confirmed cases of infection with severe acute respiratory syndrome coronavirus 2 (SARS-CoV-2) among employees at a regional hospital in the province. Five of the six cases (83%) had travelled to Boracay island. This outbreak was reported through the national Event-Based Surveillance and Response System. In response, on 25 July, trainees from the Field Epidemiology Training Programme (FETP) in Northern Luzon were sent to conduct an epidemiological investigation.

The regional hospital is a Department of Health (DOH) hospital located in Bauko, Mountain Province. The hospital has 100 beds and 681 employees, including casual labourers and contractual employees. It is the referral hospital for Mountain Province and neighbouring provinces. During the SARS-CoV-2 pandemic, the hospital accommodated asymptomatic to severe cases and served as a step-down facility from the infectious disease pavilion. The hospital also has a SARS-CoV-2 molecular laboratory, capable of running 192 tests at a time and providing routine testing for employees. Routine testing for SARS-CoV-2 is done every 15 days.

When the outbreak was reported in July 2022, Mountain Province was in Alert Level 1 due to a decrease in SARS-CoV-2 cases and successful roll-out of vaccinations; at Alert Level 1, travel within and outside of the province is permitted as well as 100% operational capacity in all establishments, provided the minimum public health standards are observed.

To promote better well-being and mental health among its employees after the SARS-CoV-2 pandemic, the hospital’s management planned team-building activities outside of the province. The activities were held on Boracay island, in Malay, Aklan, during 3–21 July 2022, and planned for Bolinao, Pangasinan, during succeeding weeks; each group attended activities for 4 days. The Boracay group was divided into three teams and the Bolinao group into two teams. The Boracay group was the first to travel.

Boracay is a small island in the Western Visayas Region known for its resorts and beaches. It was at Alert Level 1 at the time of the outbreak, and an increase in the number of foreigners visiting Boracay in July was reported from the Malay Tourism Office. On 14 July, Aklan reported 43 active cases of SARS-CoV-2 infection, including five from the municipality of Malay which includes Boracay.

## Methods

The FETP trainees conducted a descriptive epidemiological analysis to investigate confirmed cases of SARS-CoV-2 infection among staff of the regional hospital in Mountain Province, Philippines. The definition of a confirmed SARS-CoV-2 case used in this study was based on the DOH memorandum order dated 7 March 2022 and included:

any individual, irrespective of the presence or absence of clinical signs and symptoms, who had SARS-CoV-2 infection confirmed by a test conducted at the national reference laboratory, a subnational reference laboratory or a DOH-licensed SARS-CoV-2 testing laboratory, or a combination of these; OR
any suspected or probable case of SARS-CoV-2 infection who tested positive using an antigen test in areas with outbreaks or in remote settings where reverse transcription–polymerase chain reaction (RT–PCR) is not immediately available, provided that the antigen test satisfies the recommended minimum regulatory, technical and operational specifications set by the Health Technology Assessment Council.

The line list of positive cases and case investigation forms for hospital staff were obtained from the provincial database of SARS-CoV-2 cases. From this, a separate line list of confirmed SARS-CoV-2 cases among hospital staff was created.

To gather additional information, a guided questionnaire was used to conduct interviews with key informants, including the head of the human resources department, team leaders and coordinators of teams two and three from the team-building activities (the leader of team one was unavailable), and the Health Education and Promotions Officer who joined the team-building activities on Boracay. The purpose of these interviews was to identify the activities undertaken and practices observed during the team-building that may have contributed to the spread of SARS-CoV-2 among hospital staff.

## Results

**Fig. 1:**
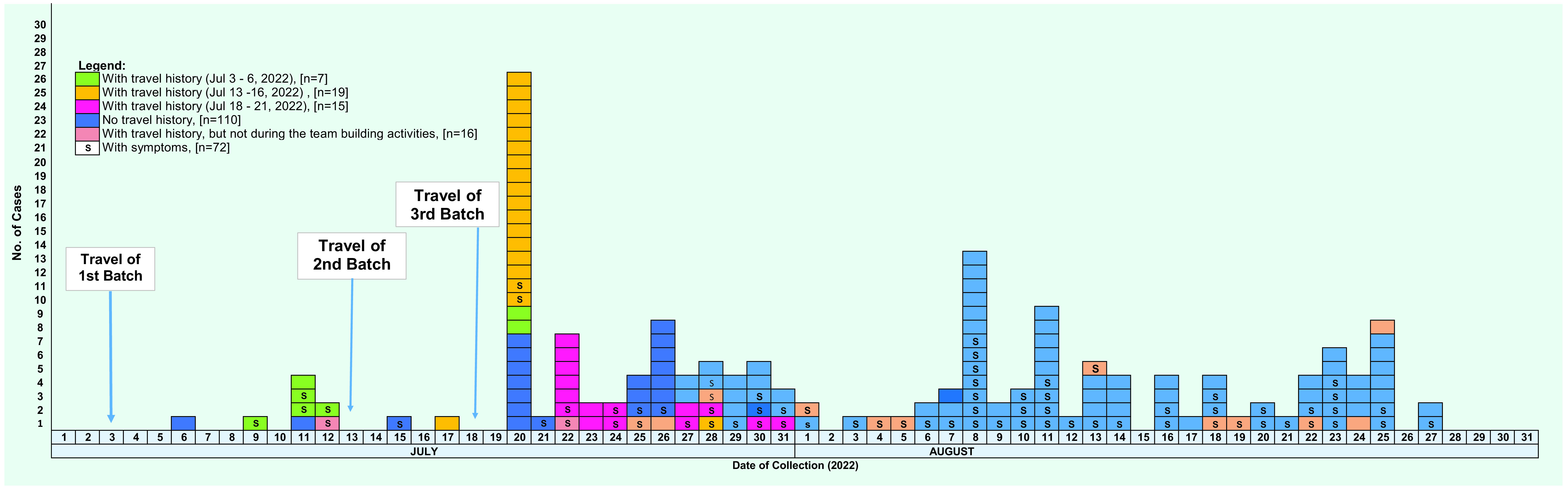
Confirmed cases of severe acute respiratory syndrome coronavirus 2 in a regional hospital, by date of specimen collection (N = 167), Philippines, 1 July–31 August 2022

From 6 July to 31 August 2022, 167 personnel and interns working at the regional hospital tested positive for SARS-CoV-2. Cases were continually detected during this period, with a peak in the number of cases on 20 July (**Fig. 1**).

[**insert** Figure 1]

Of the cases, 57 (34%) had a history of travel, and 41 (25%) had travelled to Boracay to attend the team-building activities. Among these cases, 19 (46%) were from the second group of attendees (13–16 July). Most cases (*n* = 96, 57%) were asymptomatic, and most of those who had travelled to Boracay were asymptomatic (27/41, 66%). Altogether, 96% (69/71) of cases with symptoms had mild illness. All cases recovered.

The first reported case worked in the surgery department of the hospital and was detected through routine employee testing on 6 July. The hospital reported that the case had travelled to a nearby municipality to attend a social gathering 4 days before testing. No confirmed case had been reported among hospital staff for 2 months before this case. The second case occurred in a nurse working in the outpatient department; this case was also detected during scheduled routine testing and was swabbed 3 days after returning from Boracay (**Fig. 1**).

**Fig. 2:**
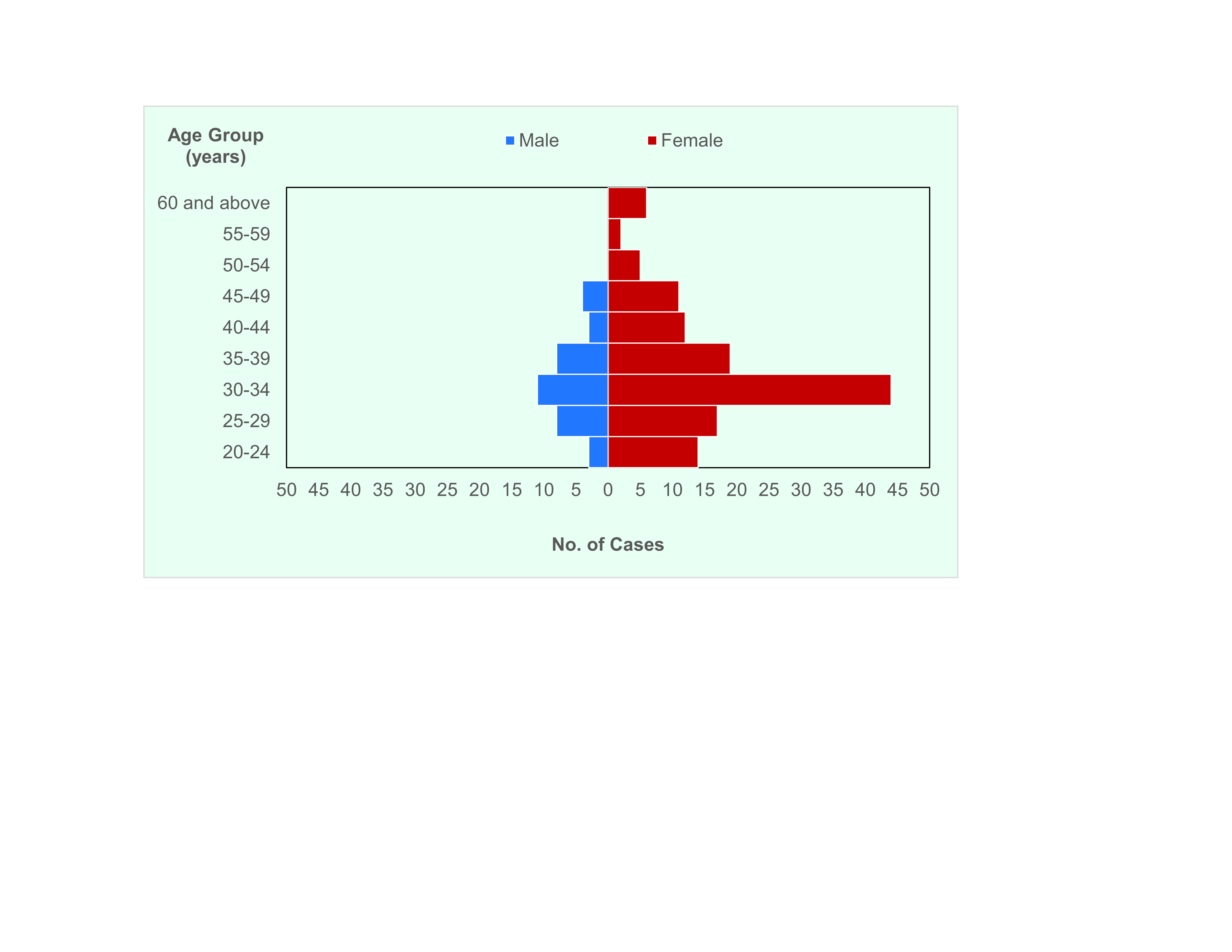
Confirmed cases of severe acute respiratory syndrome coronavirus 2 in a regional hospital, by age group and sex (N = 167), Philippines, 1 July–31 August 2022

The age range of cases was 21–64 years (median: 32 years), with the most affected age group being those aged 30–34 years (**Fig. 2**). The majority of cases occurred among females (130, 78%). Nurses accounted for the highest number of cases (60, 36%), followed by physicians (23, 14%), administrative assistants (11, 7%) and nursing attendants (8, 5%), with administrative aides, medical technologists, medical technologist interns and radiology technologists accounting for 7 cases each (4% each). The remaining cases occurred among a range of other hospital staff. Health workers were affected across all levels and wards in the hospital, and both front-line and non-front-line health workers were equally affected. Some of the cases worked in multiple areas of the hospital. All cases were fully vaccinated and had received at least one booster dose.

[**insert** Figure 2]

### Key informant interviews

Staff were not required to be tested before travel; instead, attendees only had to report that they were asymptomatic. Safety briefings were conducted before and after each activity on Boracay, and team leaders were assigned as monitors. All activities were held at the beach, and meals were served buffet style to groups in an area with good ventilation and with two to three individuals per table. Two to three participants were accommodated in each room, in which there were single beds; some two-bed rooms had an additional mattress so three people could be accommodated. The bedrooms were air-conditioned. During free time, participants visited souvenir shops and fast food centres, and some went drinking at night. It was reported that the attendees did not observe physical distancing and did not wear masks while out drinking and during water sports.

### Control measures

Several control measures were implemented by the regional hospital after the first six cases were reported. Swabbing of all employees every 15 days continued, with mandatory testing for the last team upon their return from travel. Cases were advised to isolate in their homes and were allowed to return to duty after completing 5 days’ isolation from the date of onset of symptoms or testing, as certified by their safety officer. Members of the second team, who were not scheduled for routine testing on returning from travel, were asked to undergo testing. Hospital management postponed the later travel to Bolinao. Management also strengthened their contingency plans for outbreaks at the hospital, which included promoting stricter observance of minimum public health standards in the facility at all times.

## Discussion

This outbreak highlights the impact of travel and team-building activities on the transmission of SARS-CoV-2 among health-care workers. The peak of cases among hospital staff occurred on 20 July 2022, which coincided with the team-building activities on Boracay. Similar findings have been reported in a study conducted in Petaling District, Malaysia, where the authors found that social gatherings were a significant contributor to the spread of SARS-CoV-2 among health-care workers. (*1*) This outbreak also suggests that transmission of SARS-CoV-2 among health-care workers can occur not only through direct exposure to patients in a ward but also through contact with other staff members outside of the hospital. This is consistent with previous studies, such as that of the SARS-CoV-2 outbreak in Tasmanian health-care settings, which demonstrated that transmission among health-care workers can occur outside of patient care areas. (*2*)

Health-care workers are vulnerable to SARS-CoV-2 transmission not only directly from patients but also from other staff after patient care. Ongoing transmission in the hospital may have also contributed to this outbreak as evidenced by the detection of cases with no travel history. Hospital staff working in close proximity while infectious may have unknowingly contributed to the spread of SARS-CoV-2 in the hospital. (*3*) Our investigation revealed that some cases worked in multiple areas of the hospital, which made transmission between and among staff possible. (*1*) This highlights the importance of observing physical distancing and other public health measures, especially in health-care settings where staff may work in multiple areas and come into contact with a larger number of colleagues and patients. Unrecognized asymptomatic and presymptomatic infections might also contribute to transmission in these settings. (*4*, *5*)

The majority of health-care workers who travelled were asymptomatic upon detection, and this may be attributed to their vaccination history. All cases were vaccinated, and vaccinated individuals are most likely to be asymptomatic. (*6*) Vaccination has a substantial impact on reducing the incidence of coronavirus disease, hospitalizations and deaths, especially among vulnerable individuals with comorbidities and risk factors associated with severe disease. (*7*) Fully vaccinated individuals also have fewer days during which they are symptomatic and have less severe illness. (*8*) Most of the symptomatic cases had only mild signs and symptoms.

This study underscores the need for regular testing of health-care workers, especially before and after travel or team-building activities. In this investigation, the hospital had its own molecular laboratory so it is capable of testing staff regularly. Regular testing will help identify possible sources of infection and prevent further transmission. It is also important for health-care workers who test positive to be vigilant in identifying and reporting close contacts, as this will facilitate contact tracing and prevent further spread of the virus.

This study is only descriptive and thus is limited in its ability to determine risk factors. This limitation and the absence of testing before travel make it difficult to determine whether transmission existed in the hospital before the detection of the first case. Additionally, close contacts were not disclosed, so the study was not able to trace secondary and tertiary transmission. Despite these limitations, the study was able to provide further evidence of the risk of transmission between health-care workers outside of health-care facilities.

Therefore, it is likely that the travel to and attendance at the team-building activities contributed to this outbreak of SARS-CoV-2 among the staff and interns at this regional hospital. Not observing minimum public health standards during the activities – such as physical distancing and avoiding direct contact during meals and drinking sessions – likely contributed to transmission.
